# Long-term outcome (28–40 years) after correction of leg length discrepancy through permanent epiphysiodesis

**DOI:** 10.1186/s10195-025-00895-2

**Published:** 2025-12-16

**Authors:** Andrea Laufer, Paula Swoboda, Georg Gosheger, Jan Duedal Rölfing, Adrien Frommer, Gregor Toporowski, Max Masthoff, Robert Roedl, Bjoern Vogt

**Affiliations:** 1 Pediatric Orthopedics, Deformity Reconstruction and Foot Surgery, Muenster University Hospital, Muenster, Germany; 2 General Orthopedics and Tumor Orthopedics, Muenster University Hospital, Muenster, Germany; 3 Children’s Orthopedics and Reconstruction, Aarhus University Hospital, Aarhus, Denmark; 4 Clinic for Radiology, Muenster University Hospital, Muenster, Germany

**Keywords:** Leg length discrepancy, Permanent epiphysiodesis, Phemister technique, Long-term results, Knee joint, Angular deformity, Osteoarthritis

## Abstract

**Background:**

Permanent epiphysiodesis (pED) according to Phemister is an established treatment for leg length discrepancies (LLD) but has largely been replaced by less invasive techniques. Nevertheless, modern pED procedures based on the Phemister principle are still widely used in paediatric orthopaedics for LLD correction and treatment of tall stature. However, the long-term effects of pED on the knee joint remain unclear. This study aimed to evaluate the long-term outcomes of Phemister pED, specifically assessing secondary alterations in knee joint morphology and the incidence of pre-mature osteoarthritis. A clearer understanding of these sequelae may help guide treatment decisions in paediatric orthopaedic care.

**Materials and methods:**

A retrospective review of our institution’s longitudinal database identified 75 patients who underwent Phemister pED for LLD between 1980 and 2006. Of these, 20 patients met inclusion criteria and were available for long-term evaluation. Their clinical and radiographic outcomes were compared with those of an age- and sex-matched control cohort of ten untreated individuals. Clinical and radiographic assessments included LLD, mechanical axis deviation, joint orientation angles, central knee anatomy and osteoarthritis grading. Patient-reported outcomes were evaluated using the Oxford Knee Score (OKS), EQ-5D-3L and Knee Injury and Osteoarthritis Outcome Score (KOOS).

**Results:**

The median follow-up was 37 years (interquartile range 33–39). The mean pre-operative LLD of 2.8 cm (standard deviation (SD) 0.7) was reduced to 1.1 cm (SD 0.6) at last follow-up, although 55% of patients had residual LLD > 1 cm. No relevant differences in joint alignment or central knee anatomy were found between patients and controls. Mild knee osteoarthritis (Kellgren–Lawrence grade 1) was observed in two patients and none in controls. Patient-reported outcomes showed lower OKS and EQ-5D-3L scores in the pED group, although KOOS scores were similar.

**Conclusions:**

Phemister pED showed satisfactory long-term results for LLD correction, without secondary angular or intra-articular deformities or relevant knee osteoarthritis. Despite slightly lower function and more discomfort, findings support the use of modern pED techniques based on the Phemister principle. This is especially relevant for elective indications such as tall stature. Further comparative studies with percutaneous methods remain necessary to confirm these observations.

*Level of evidence* Level IV, therapeutic study.

**Supplementary Information:**

The online version contains supplementary material available at 10.1186/s10195-025-00895-2.

## Introduction

Permanent epiphysiodesis (pED) is a well-established treatment approach for the correction of leg length discrepancies (LLD) between 2 and 5 cm in skeletally immature patients. Traditionally, pED has been performed according to the technique originally described by Phemister [1], showing satisfactory results regarding leg length equalisation [2, 3]. With the advent of percutaneous pED techniques, facilitated through the introduction of intraoperative fluoroscopy, Phemister pED has been widely abandoned [3]. Nonetheless, several modern pED techniques are based on the Phemister principle and are commonly used to correct LLD [2–4]. For instance, a novel bone trephine is applied for the correction of LLD and the treatment of tall stature by controlled removal of a cylindrical bone segment containing the central part of the growth plate, which is then reinserted in a 90-degree rotated position to induce the pre-mature closure of the growth plate, in line with the Phemister principle [3]. More recently, the bilateral application of the procedure has been established for treatment of excessive body height [5]. Even though tall stature is not a pathological condition per se and generally does not require specific treatment, families of skeletally immature individuals with tall stature who report a psychosocial impact and have a strong treatment desire may be counselled on available treatment options [6]. Since the application of high-dose sex steroids has been associated with severe long-term effects [7, 8], there is an increasing demand for alternative surgical treatment [5, 6, 9]. Even though short-term results indicate that pED based on the Phemister principle is associated with few complications [5], it still represents an invasive procedure mostly performed in healthy children. The long-term outcome—in particular, concerning knee morphology and the risk of early-onset osteoarthritis—remains unclear. While the effectiveness and safety of implant-mediated (hemi-)epiphysiodesis around the knee has been partially elucidated regarding alterations of joint configuration [10, 11], there are no long-term studies evaluating the effects of open pED techniques. Owing to this lack of long-term assessments, it remains questionable whether the satisfactory short-term treatment outcome of pED justifies the continued application. To facilitate benefit–risk assessment of this procedure, this study aims to evaluate the long-term outcomes of pED, specifically regarding knee joint morphology, development of pre-mature osteoarthritis, knee function and LLD reduction.

## Patients and methods

### Study design and setting

In this cohort study, clinical and radiological results of a historical cohort of patients surgically treated with Phemister pED to correct LLD were evaluated and compared with those of an untreated control group without preceding knee operations or pathologies. The study was based on a previous retrospective analysis of short-term results of 178 patients who were surgically treated between 1980 and 2006 with Phemister and percutaneous pED for treatment of LLD and angular deformities [12].

### Participants

**Fig. 1:**
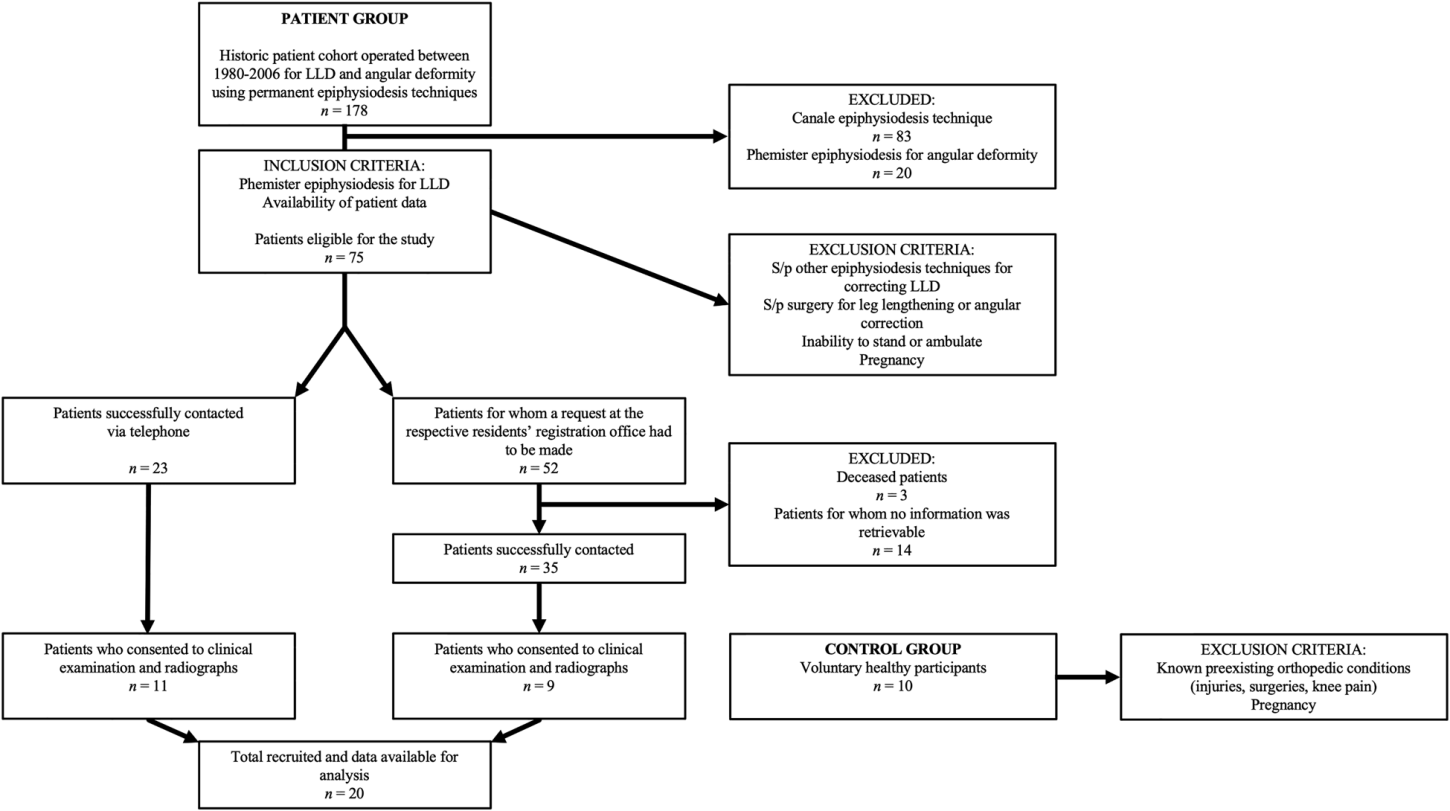
This STROBE diagram details the inclusion and exclusion criteria for the study cohort. *LLD* leg length discrepancy, *S/p* status post

Of the 178 patients included in the previous study, 75 patients were treated for LLD by Phemister pED, of whom 20 patients fulfilled the inclusion criteria and were ultimately available for analysis (Fig. 1). In most of these patients, LLD originated in pathologies of the shorter, untreated leg, which thus could not be used for comparison. Therefore, a control group was retrospectively enrolled after the identification of the eligible patient cohort to ensure a matching for age and sex distribution. Its purpose was to provide a reference to the general population and assess whether pathologies occur more frequently in Phemister pED patients than in individuals without previous knee pathology. We chose a 2:1 ratio of patients to controls to maximise statistical power while maintaining strict comparability, given the limited number of eligible controls in the relevant age range.

### Demographics

Complete data sets of clinical and radiological evaluations and questionnaires were obtained for all 20 patients and all 10 controls. The two groups did not vary regarding main demographics (Table 1). The mean age at examination was 49 years (standard deviation (SD) 4.6) in the patient group and 47 years (SD 5.4) in the control group. The distribution of sex was 8/20 (40%) female and 12/20 (60%) male in the patient group and 5/10 (50%) female and 5/10 (50%) male in the control group.

**Table 1:** Baseline demographics of all study participants (*n* = 30)

	Epiphysiodesis (*n* = 20)	Control (*n* = 10)	*p*-Value
Age (years)			0.28
Mean (SD)	49 (4.6)	47 (5.4)	
Sex (no. of patients (%))			0.705
Male	12 (60)	5 (50)	
Female	8 (40)	5 (50)	

SD, standard deviation; no., number

The median follow-up time was 37 years (interquartile range (IQR) 33–39) in the patient cohort, with a mean chronological age at surgery of 13.9 years (SD 1.4). The pre-operative skeletal age was assessed using the Greulich and Pyle method and rounded to full years [13]. The mean skeletal age at operation was 13.0 years (SD 0.5) in female and 14.6 years (SD 0.5) in male patients. The mean pre-operative LLD was 2.8 cm (SD 0.7) (Table 2).

**Table 2:** Demographics of patients with epiphysiodesis (*n* = 20)

	Total	Male (*n* = 12)	Female (*n* = 8)
Chronological age at operation (years)			
Mean (SD)	13.9 (1.4)	14.8 (0.7)	12.6 (1.23)
Minimum	11.3	13.5	11.3
Maximum	16.4	16.4	14.4
Skeletal age at operation (years)			
Mean (SD)	14 (0.5)	14.6 (0.5)	13 (0.5)
Minimum	15	14	12
Maximum	12	15	14
Years since operation (years)			
Median (IQR)	37 (33–39)		
Location of the operated physis (no. of patients (%))			
Distal femur	3 (15)		
Proximal tibia	4 (20)		
Distal femur + proximal tibia	13 (65)		
LLD pre-operative clinical (cm)			
Mean (SD)	2.8 (0.7)		
Minimum	1.3		
Maximum	4.3		
Data missing (no. of patients)	2		
LLD at latest follow-up (cm)			
Mean (SD)	1.1 (0.6)		
Minimum	0.1		
Maximum	2.3		
Rating of LLD at last follow-up (no. of patients (%)			
Good result (< 1.5 cm)	13 (65)		
Fair result (1.5–2 cm)	6 (30)		
Poor result (> 2 cm)	1 (5)		
LLD reduction* (cm)			
Mean (SD)	1.7 (1.1)		
Minimum	−0.2		
Maximum	4.2		
Data missing (no. of patients)	2		

SD, standard deviation; IQR, interquartile range; LLD, leg length discrepancy; no., number

*Difference of pre-operative LLD and LLD at last follow-up

In 13/20 legs (65%), both the distal femur and the proximal tibia were treated by Phemister pED, while in 7/20 (35%) only one physis was surgically treated, with 3/20 (15%) operations on the distal femur and 4/20 (20%) on the proximal tibia.

The etiologies of LLD are given in Table 3; the original patient data are presented in Table 4.

**Table 3:** Aetiology of patients with epiphysiodesis (*n* = 20)

Etiology	No. of patients (%)
Congenital LLD	12 (60)
Hemi-hypertrophy	5 (25)
Hip deformity*	7 (35)
Secondary LLD	4 (20)
Post-traumatic	4 (20)
Idiopathic LLD	4 (20)

no., number; LLD, leg length discrepancy

*Hip deformity includes developmental hip dysplasia and Perthes’ disease

**Table 4:** Original data set of patients with epiphysiodesis (*n* = 20)

Case no.	Sex	Skeletal age at operation (years)	Follow-up time (years)	Etiology*	Pre-operative LLD (cm)	Current LLD (cm)	LLD reduction (cm)†
1	f	13	38	Hip deformity	3.2	0.5	2.7
2	m	15	37	Idiopathic	1.3	1.0	0.3
3	f	13	28	Hemi-hypertrophy	No data	1.5	No data
4	m	14	33	Idiopathic	2.8	0.8	2.0
5	m	14	36	Fracture tibia + fibula	3.0	1.3	1.7
6	m	14	35	Hemi-hypertrophy	2.1	2.3	−0.2
7	m	15	40	Fracture femur	2.5	1.2	1.3
8	m	15	39	Idiopathic	2.5	2.0	0.5
9	f	13	28	Hemi-hypertrophy	No data	0.6	No data
10	m	15	40	Idiopathic	2.5	0.5	2.0
11	m	14	35	Hip deformity	2.2	0.6	1.6
12	f	13	40	Hip deformity	3.5	0.6	2.9
13	m	15	38	Hip deformity	4.3	0.1	4.2
14	f	13	33	Hip deformity	4.0	1.6	2.4
15	m	15	37	Fracture femur	2.5	0.2	2.3
16	f	13	29	Hip deformity	3.6	1.8	1.8
17	f	14	37	Fracture tibia + fibula	1.9	1.4	0.5
18	m	14	28	Hemi-hypertrophy	2.8	1.7	1.1
19	f	12	40	Hip deformity	3.0	1.6	1.4
20	m	15	37	Idiopathic	2.4	0.6	1.8

no., number; LLD, leg length discrepancy; m, male; f, female

*Hip deformity includes developmental hip dysplasia and Perthes’ disease

^†^Difference of pre-operative LLD and LLD at last follow-up

### Surgical technique

**Fig. 2:**
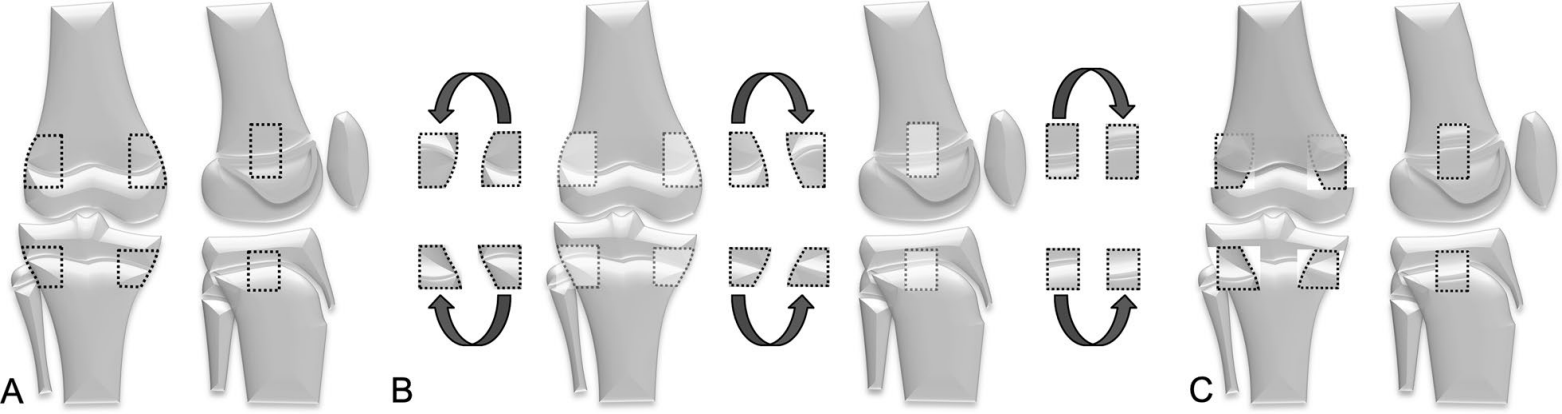
Illustration of the traditional Phemister epiphysiodesis technique

Phemister pED comprises the extraction of a rectangular block of cortical bone containing the peripheral physis with adjacent metaphyseal and epiphyseal bone [1]. Subsequently, the growth plate is chiselled out with a depth of approximately 1 cm before the bone block is reinserted after 180-degree rotation (Fig. 2). Pre-mature epiphysiodiaphyseal fusion is induced by the formation of the bony bridge. The procedure is executed on the lateral and medial side of the growth plates of the distal femur and/or proximal tibia of the longer leg to achieve growth arrest.

### Outcome parameters

All patients and controls were examined following the same criteria of clinical examination, radiographic evaluation and questionnaires. In patients, questions and examinations were performed regarding the operated leg. In controls, one leg was randomly chosen before examination.

All pre-operative radiological parameters were measured as part of the previous retrospective analysis of short-term results using the original, non-digital radiographs.

For follow-up radiographic examination of patients and radiographic evaluation of controls, a long-standing anterior–posterior image and one lateral image of the knee were conducted. Radiological measurements were carried out on calibrated radiographs using the PACS^®^ system (GE Healthcare, Chicago, IL, USA) and the postprocessing software TraumaCad^®^ (Brainlab, Munich, Germany).

To evaluate the LLD, the distance between the two horizontal tangents at the top of the femoral heads was measured on an anteroposterior long-standing radiograph. A pathological LLD was defined as a distance of > 1 cm. Success of surgical LLD reduction was defined according to the criteria set by Kemnitz et al., deeming residual LLD < 1.5 cm a good result, 1.5–2 cm a fair result and LLD > 2 cm a poor result [14].

**Fig. 3:**
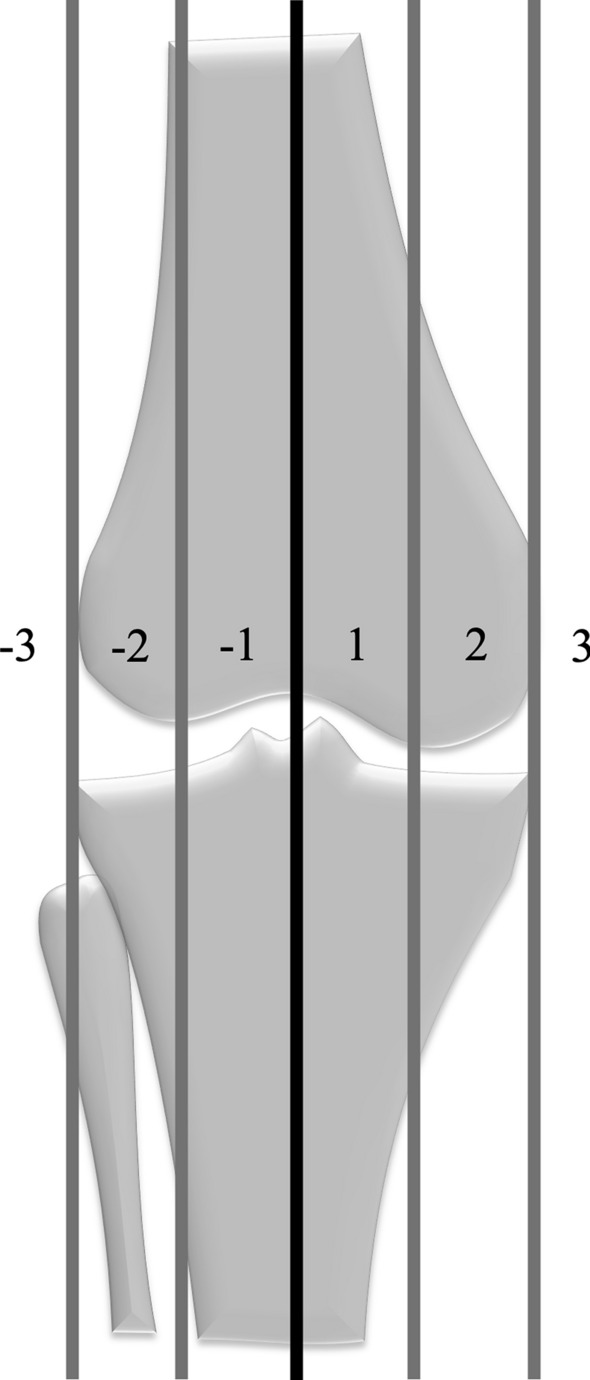
Zones of the mechanical axis. Zones ± 2 and ± 3 were defined as pathological axis deviations

**Fig. 4:**
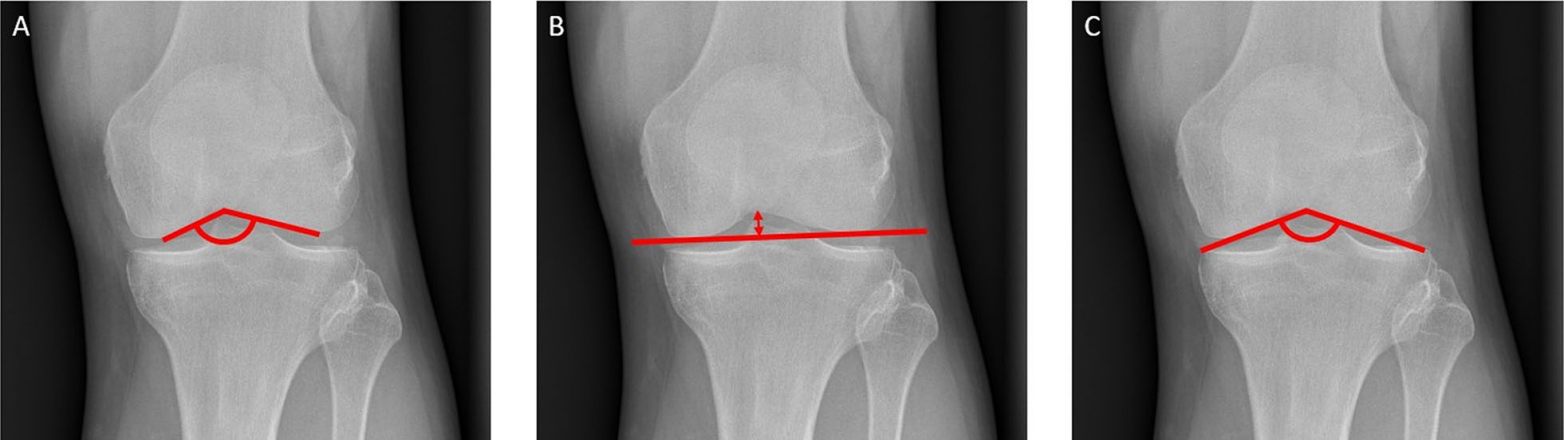
Measurement of the femoral floor angle (**A**), the femoral notch intercondylar distance (**B**) and the tibial roof angle (**C**)

The effect of the intervention on knee joint morphology was investigated regarding frontal alignment and intra-articular parameters. Mechanical axis deviation (MAD) was categorised as previously suggested, with a MAD outside zone 1 or −1 classified as a pathological axis deviation (Fig. 3) [15]. Joint orientation was assessed using the established parameters mechanical lateral distal femur angle, medial proximal tibia angle, posterior distal femur angle and proximal posterior tibia angle [16]. For the assessment of central knee anatomy and intra-articular deformities, the femoral floor angle, femoral notch intercondylar distance and the tibial roof angle were evaluated [10, 11, 17] and compared with established reference values (Fig. 4A–C) [18].

Regarding the assessment of osteoarthritis of the knee joint, the Kellgren–Lawrence classification [19] was employed on anteroposterior radiographs of the knee of patients and controls. To evaluate the patients’ clinical outcome and compare it with healthy controls, we included objective as well as subjective parameters. Range of motion (ROM) and knee joint stability were clinically determined. For the subjective evaluation of the clinical outcome, the Oxford Knee Score (OKS) was used to evaluate knee-related health, function and pain [20]; the EQ-5D-3L was employed to assess the quality of life of patients [21]; and the Knee Injury and Osteoarthritis Outcome Score (KOOS) was used to evaluate knee-related function, pain and quality of life [22].

### Statistical analysis

For non-metric categorial nominal parameters, descriptive statistics were performed using cross tables and compared using Fisher’s exact test for 2 × 2 cross tables and *χ*^2^ test for all larger cross tables. For normally distributed metric parameters, mean values were stated with standard deviation (SD) in parentheses. Mean values were compared using the *t*-test for independent samples. For non-normally distributed metric data, median values were stated with the IQR and presented as 25th–75th percentile. Median values were compared using the Mann–Whitney *U* test. The Wilcoxon signed-rank test was used to compare with related samples. A 95% confidence interval was chosen, and the significance level was set at *α* < 0.05.

Subgroup analysis by gender was not performed because the gender groups were too small.

Statistics were conducted using IBM SPSS Statistics Version 29.0.2.0 (IBM, NYC, USA).

## Results

### Knee joint morphology and osteoarthritis

Patients who received Phemister pED did not show a relevant pathological change in frontal or sagittal alignment, nor in intra-articular joint orientation when compared with controls. The mean MAD in patients was 9.9 mm (SD 5.7) compared with 6.7 mm (SD 3.6) in controls. Coronal malalignment was not found in either group (Table 5).

**Table 5:** Radiological parameters in all study participants (*n* = 30)

	Reference	Epiphysiodesis (*n* = 20)	Control (*n* = 10)	*p*-Value
mLDFA (degree)	85–90			0.914
Mean (SD)		87.7 (2.2)	87.9 (1.9)	
MPTA (degree)	85–90			0.812
Mean (SD)		88.5 (2.1)	88.4 (2.3)	
PDFA (degree)	79–87			1
Mean (SD)		83.9 (2.6)	84.5 (2.5)	
PPTA (degree)	77–84			0.432
Mean (SD)		80.6 (2.6)	81.1 (1.3)	
MAD (mm)				0.1
Mean (SD)		9.9	6.7	
Stevens zone (no. of patients (%))				1
1 or −1		20	10	
2 or −2		0	0	
3 or −3		0	0	
FFA (degree)	130–154			0.495
Mean (SD)		143.4	144.6	
FNID (mm)	5–11			0.812
Median (IQR)		7.5 (7–8)	8.0 (7–8)	
TRA (degree)	134–154			0.137
Mean (SD)		145.7 (4.1)	147.8 (2.1)	

mLDFA, mechanical lateral distal femur angle; SD, standard deviation; MPTA, medial proximal tibia angle; PDFA, posterior distal femur angle; PPTA, proximal posterior tibia angle; MAD, mechanical axis deviation; no., number; FFA, femoral floor angle; FNID, femoral notch intercondylar distance; IQR, interquartile range; TRA, tibial roof angle

Knee joint orientation angles outside reference values were found in 5/20 (25%) patients and in 2/10 (20%) controls. The mean values of the individual joint orientation angles did not differ between the groups (Table 5).

The radiological parameters assessing central knee joint anatomy did not differ between patients and controls, neither were there any pathological values regarding reference values (Table 5).

**Fig. 5:**
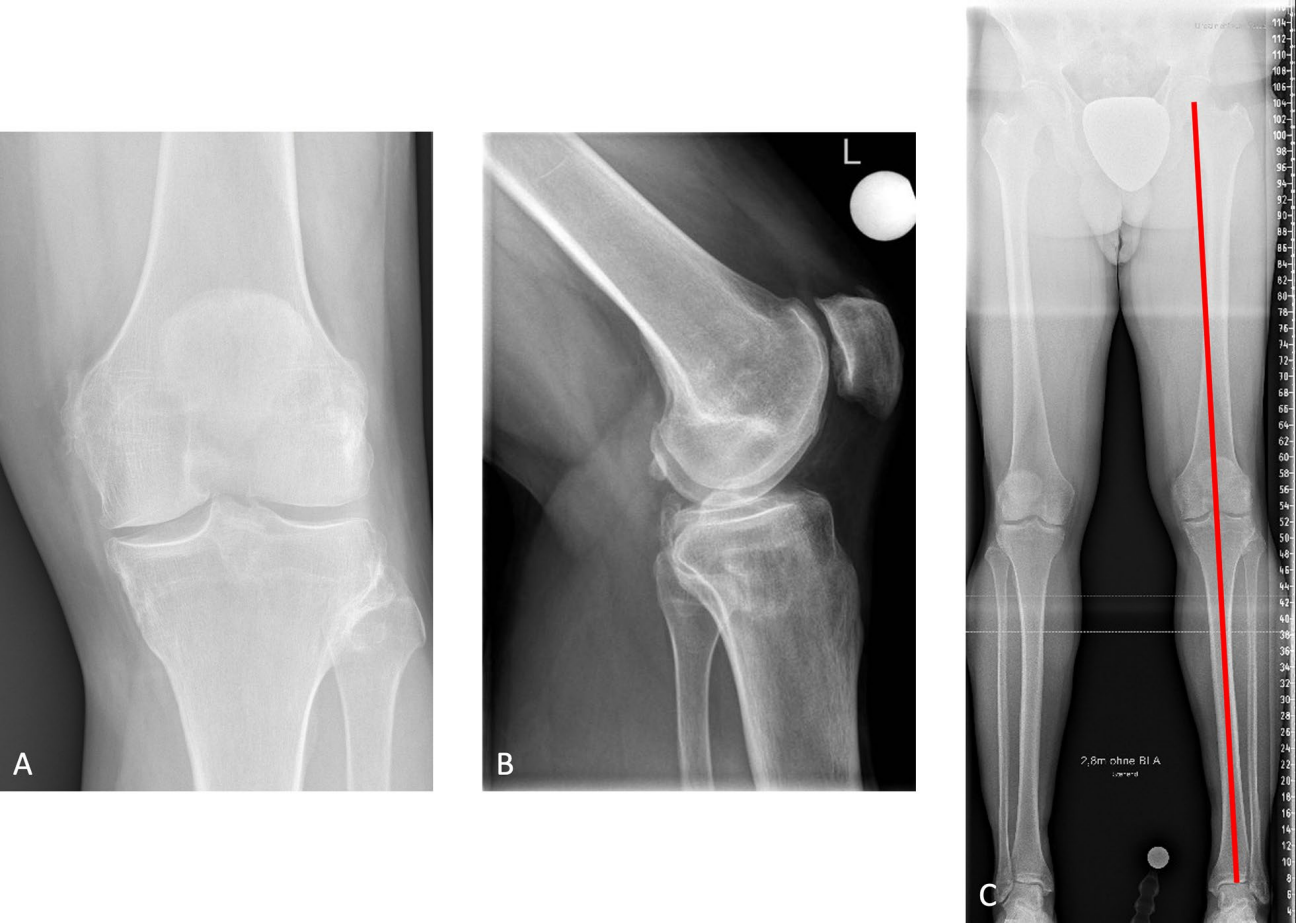
Anteroposterior (**A**) and lateral (**B**) radiographs of 52-year-old male, 37 years after Phemister epiphysiodesis of the left distal femur and proximal tibia, showing osteoarthritis of the knee joint Kellgren and Lawrence grade I but regular intra-articular joint anatomy and physiological limb alignment (**C**)

Patients did not show a relevantly higher rate of osteoarthritis of the knee than healthy controls. Osteoarthritis of the knee joint Kellgren–Lawrence grade 1 was observed in 2/20 (10%) patients (Fig. 5), with no signs of osteoarthritis in the contralateral knee, whereas all controls showed Kellgren–Lawrence grade 0. The first patient with osteoarthritis showed a pathological MAD of 19 mm towards varus as well as a remaining LLD of 1.6 cm of the treated leg. The LLD resulted from bilateral hip dysplasia, which led to bilateral osteoarthritis of the hip and a total hip arthroplasty in the untreated leg in adulthood. The second patient showed no pathological remaining LLD or joint angles.

### Knee function and patient satisfaction

Patients who underwent Phemister pED reported more knee-specific discomfort than healthy controls. However, this could not be objectified, as no relevant restrictions of ROM were found in patients clinically.

In total, 8/20 (40%) patients reported knee-specific discomfort in the surgically treated leg, while none in the control group reported any discomfort (*p* = 0.029). Of those with discomfort, four out of eight patients reported pain after longer periods of physical activity; however, three out of four exhibited this pain in both knees. Other discomfort declared was limited mobility in two out of eight, hypoesthesia in one out of eight and knee crepitation in one out of eight (Table 6). There were no notable restrictions of ROM, nor did the ROM relevantly differ between the two groups (Table 7).

**Table 6:** Clinical data of all study participants (*n* = 30)

	Epiphysiodesis (*n* = 20)	Control (*n* = 10)	*p*-Value
Clinical complaints (no. of patients (%))			0.029
Yes	8 (40)	0 (0)	
No	12 (60)	10 (100)	
Type of complaints (no. of patients (%))			
Stress pain	4 (50)		
Limited mobility	2 (25)		
Hypesthesia	1 (12.5)		
Knee crepitation	1 (12.5)		

no., number

**Table 7:** Knee joint range of motion in all study participants (*n* = 30)

	Reference	Epiphysiodesis (*n* = 20)	Control (*n* = 10)	*p*-Value
Flexion (degree)	120–150			0.2
				14
Median (IQR)		140 (140–150)	150 (137–150)	
Neutral (degree)	0			0.846
Median (IQR)		0 (0)	0 (0)	
Extension (degree)	5–10			
Median (IQR)		5 (5–10)	5 (5–10)	0.530

IQR, interquartile range

Regarding the subjective evaluation of knee-related health, patients tended to show lower scores in questionnaires than controls.

The OKS total score was lower in patients than in controls, with a median score of 46/48 (44.0–47.0) in patients and 48/48 (47.8–48.0) in controls (*p* = 0.003). Both the OKS pain and the function subscales showed lower scores in patients than in controls (pain: *p* = 0.005; function: *p* = 0.024).

The EQ-5D-3L total scores for each item were lower in patients than in controls (Supplementary Table 2). The ‘health today’ score subscale was lower in patients, with a median of 80 (70–86) and 90 (84–95) in controls (*p* = 0.012).

The KOOS total scores in each category were lower in patients (Supplementary Table 3).

### LLD reduction

The mean LLD in patients was higher than in controls, with 1.1 cm (SD 0.6) in patients and 0.3 cm (SD 0.2) in controls (*p* < 0.001). However, the majority of patients showed a good result regarding residual LLD. A pathological LLD (> 1 cm) was found in 11/20 (55%) of patients, while there was none in the control group (Table 8). In total, 9 out of those 11 patients had received Phemister pED both at the distal femur and the proximal tibia, while in 2 patients, the pED was performed solely at the proximal tibia. A good result of residual LLD (< 1.5 cm) was achieved in 13/20 (65%) patients, a fair result (1.5–2 cm) in 6/20 (30%), a poor result (> 2 cm) in 1/20 (5%) patients. With a mean pre-operative LLD of 2.8 cm (SD 0.7), the difference was reduced by the surgical treatment by a mean of 1.7 cm (SD 1.1) (Table 7).

**Table 8:** Leg length discrepancy in all study participants (*n* = 30)

	Epiphysiodesis (*n* = 20)	Control (*n* = 10)	*p*-Value
LLD (cm)			< 0.001
Mean (SD)	1.1 (0.6)	0.3 (0.2)	
Minimum	0.1	0	
Maximum	2.3	0.7	
Median (IQR)	1.1 (0.6–1.6)	0.2 (0.08–0.4)	
25th percentile	0.6	0.075	
75th percentile	1.6	0.4	
LLD (no. of patients (%))			0.004
< 1 cm	9 (45)	10 (100)	
> 1 cm	11 (55)	0 (0)	

LLD, leg length discrepancy; SD, standard deviation; IQR, interquartile range; no., number

In 2/20 (10%) patients, the initially shorter leg was longer at last follow-up examination owing to overcorrection; however, only one patient’s LLD was > 1 cm.

## Discussion

After the introduction in 1933, Phemister pED has been employed over several decades, with most studies reporting satisfactory short-term results regarding the equalisation of leg length [4, 23]. However, with the advent of percutaneous [24, 25] and implant-mediated [26] epiphysiodesis techniques, Phemister pED was deemed overly invasive and, consecutively, widely abandoned [3, 27]. Temporary epiphysiodesis offers the additional advantage of conversion into a hemi-epiphysiodesis by partial implant removal to achieve guided growth in case of secondary coronal malalignment. However, another surgery for hardware removal is inevitably required. Moreover, it appears that temporary epiphysiodesis methods are less efficient in leg length equalisation while showing a higher complication rate compared with pED, in particular regarding the occurrence of secondary coronal malalignment [3, 5, 28–30]. In pED, complications such as under- or overcorrection and secondary angular deformity seem to occur equally in the Phemister technique and percutaneous epiphysiodesis [2, 23]. The latter technique, however, demands precise execution and is thus associated with an increased fluoroscopy time. Insufficient physeal ablation may lead to failure of epiphysiodesis with incomplete LLD correction and secondary malalignment [31, 32]. There have been efforts to address this issue by applying techniques such as an air physiogram, which is obtained to confirm sufficient removal of the growth plate after drilling [33]. Another approach has been the combination of percutaneous pED techniques with the Phemister principle. A recently introduced bone trephine to harvest a bone cylinder containing the central part of the growth plate is applied in a minimally-invasive manner but allows visual control of the complete extraction of the central part of the physis, hence reducing the risk of incomplete physeal ablation and consecutive coronal or sagittal malalignment [3, 5]. pED with a bone trephine appears to produce lower rates of secondary malalignment with reduced intraoperative fluoroscopy time and more favourable scarring compared with implanted-mediated temporary epiphysiodesis when bilaterally applied for the treatment of tall stature [5]. Even though LLD correction still represents the main indication for pED, in the light of the long-term adverse events which have been linked to hormone treatment, there has been an increasing demand in surgical treatment to limit excessive longitudinal growth [34, 35]. Bilateral pED of the growth plates adjacent to the knee joint has shown satisfactory outcomes in this regard [5, 35]. Nonetheless, in this specific constellation, there is a debatable indication for pED performed in healthy children under medical considerations [36]. Profound knowledge of the effects of pED on joint morphology and potential long-term sequelae are thus crucial. However, to date, there are no long-term studies that follow patients who underwent pED beyond skeletal maturity.

### Limitations

Although the study design is homogeneous in terms of indication for surgery and surgical technique, the age at the time of surgery differed and may potentially influence the treatment outcome, particularly regarding leg length equalisation. In addition, the aetiologies for LLD were diverse within our patient cohort, possibly further influencing results.

Moreover, since this study was retrospective, drawing from a patient cohort treated 28–40 years ago, the availability of patient charts and pre-operative radiographs was limited. All pre-operative radiological data were used from the previous analysis of the patient cohort and could not be verified, as the original radiographs were no longer available. However, since part of our author team conducted that analysis, we were confident in the reliability of this information. Consequently, a direct comparison of knee joint morphology pre-operatively and at last follow-up was not feasible. Since no relevant pathologies were observed at follow-up, this was considered negligible. Furthermore, some recall bias likely may have occurred, as patients who were surgically treated are considered more sensitive to noticing discomfort in their knee. Because this study acted as a voluntary check-up, patients who already experienced discomfort in the operated knee were more likely to consent to participation, which might lead to a response bias, as only one third of all contacted patients consented to participation. Furthermore, we find several biases regarding the patients’ subjective evaluation of knee-related discomfort and health-using scores. Since 16/20 (80%) of patients had non-idiopathic causes for LLD, these conditions may have acted as a confounder, resulting in increased pain, less function or less quality of life, and their impact cannot safely be separated from the impact of surgery. In addition, several patients had other conditions, such as lung cancer or ulcerative colitis, which might have contributed to lower scores across patients.

### Knee joint morphology and osteoarthritis

Secondary angular deformities due to incomplete physeal closure and consecutive asymmetrical growth are generally observed after both Phemister pED and percutaneous epiphysiodesis, with incidences ranging from 3.3% to 11% [2, 4, 14, 37]. There are no conclusive findings if angular deformities occur more often in open or percutaneous techniques. While some studies reported no deformities in Phemister pED and percutaneous epiphysiodesis [38] or similar rates across both techniques [2], others found higher rates in Phemister pED cases [37]. In the present study, neither secondary angular deformities resulting in a pathological axis deviation nor pathological parameters of central knee joint anatomy suggesting intra-articular deformity were found.

In our study, 2/20 (10%) patients exhibited a Kellgren–Lawrence osteoarthritis grade 1. One of these two patients was asymptomatic but presented a MAD of 19 mm and a residual LLD of 1.6 cm. The second patient reported swelling in the knee after strenuous activity but did not show any pathological radiological parameters. There was no verifiable association between pre-mature osteoarthritis of the knee joint and secondary deformities after epiphysiodesis. Furthermore, even though larger LLD is considered to be associated with the development of osteoarthritis of the knee [39], this observation was not made in the present study. While there was a rather large portion of patients with insufficient LLD reduction (7/20 patients), there was only 1 patient who presented LLD of > 1.5 cm and osteoarthritis of the knee joint.

### Knee function and patient satisfaction

The three questionnaires OKS, EQ-5D-3L and KOOS overall documented more pain and less function and quality of life in the patient group in comparison with the controls. While not all subscores varied relevantly, patients continuously scored worse. This aligns with the findings of the clinical examination, where 8/20 (40%) patients and no controls stated any knee-specific discomfort. While not all types of discomfort could be objectivised through clinical or radiological examination, we could confidently exclude angular deformities and restrictions in ROM as specific causes. As previously discussed, the patients’ subjective clinical outcome is subject to several limitations due to the study’s long-term retrospective design.

### LLD reduction

Several studies have stated a similar efficacy of LLD reduction comparing open Phemister pED and percutaneous techniques [2, 23]. However, reduction rates and the extent of residual LLD differed considerably amongst studies. For Phemister pED, good results (≤ 1.5 cm) were found in 67–82%, fair results (1.5–2 cm) in 3–14% and poor results (≥ 2 cm) in 11–30% [4, 14, 23]. In comparison, for percutanteous epiphysiodesis, 40–89% good results, 7–46% fair results and 4–14% poor results were found [23, 40]. When compared directly, several studies found that the Phemister technique failed to achieve adequate LLD reduction more often than percutaneous epiphysiodesis [4, 23]. In the present study, we observed 13/20 (65%) good results, 6/20 (30%) fair results and 1/20 (5%) poor result. It should be taken into consideration that our patients’ skeletal age at the time of the intervention was higher (male 14.6, female 13.0 years) than in the aforementioned studies (male 12.0–14.0, female 11.5–12.0 years) [4, 14, 23, 41], with less remaining growth potential to correct LLD. This might explain why sufficient leg length equalisation was achieved in less patients compared with other studies. Nonetheless, the present study also showed fewer poor results, with the mean residual LLD of 1.1 (0.1–2.3) cm being comparable to other studies in terms of effectiveness. Lampe et al. found a mean residual LLD of 1.4 cm after Phemister pED [41], while Timperlake et al. found a mean residual LLD of 1.5 cm after percutaneous epiphysiodesis [40].

The overcorrection of LLD as an adverse effect is frequently observed in different techniques of pED for LLD [2, 23]. Across all techniques, overcorrection rates of 0.7–10% were found, defining a LLD of > 1.5 cm as a relevant overcorrection [2, 14]. In the present study, we observed relevant overcorrection of 1.6 cm in one patient (5%). As the patient had received a total hip arthroplasty in adulthood in the untreated leg, this might be a competing factor for the presumed overcorrection.

Precise timing of the epiphysiodesis is crucial to prevent over- or undercorrection [4, 23, 31]. In the present study, initial charts and radiographs prior to surgery were no longer available, thus the correct estimation of skeletal age and timing of the intervention could not be assessed. Considering the mean chronological age at surgery, we assume that several patients received surgical treatment at an age too advanced to achieve complete leg length equalisation. Furthermore, prediction of residual growth is inaccurate in physes affected by trauma or specific congenital conditions [26], which may further influence the success of treatment.

## Conclusions

The findings of this study did not indicate a negative long-term impact of pED on the knee joint, particularly regarding the development of intra-articular deformities, angular malalignment or pre-mature osteoarthritis. Even though patients reported more knee-related discomfort than controls, this could not be clinically confirmed. Comparing the long-term results of Phemister pED in the present study with previously evaluated short-term results of both Phemister pED and percutaneous epiphysiodesis, we found a comparable outcome regarding LLD reduction and better results regarding secondary axis deviations. We thus postulate that Phemister pED produces satisfactory long-term results. Even though the traditional Phemister pED should be considered obsolete due to being overly invasive, the findings of this study may warrant the application of modern pED techniques based on the Phemister principle. However, considering the current study data, definitive conclusions on the advantages or disadvantages of Phemister pED compared with percutaneous techniques cannot be drawn. Further studies will have to investigate this aspect.

## Supplementary Information

Additional file1 (DOCX 18 KB)

## Data Availability

The datasets used and/or analysed during the current study are available from the corresponding author on reasonable request.

## Abbreviations

FFA: Femoral floor angle
FNID: Femoral notch intercondylar distance
IQR: Interquartile range
KOOS: Knee Injury and Osteoarthritis Outcome Score
LLD: Leg length discrepancy
MAD: Mechanical axis deviation
mLDFA: Mechanical lateral distal femur angle
MPTA: Medial proximal tibial angle
OKS: Oxford Knee Score
PDFA: Posterior distal femur angle
pED: Permanent epiphysiodesis
PPTA: Proximal posterior tibia angle
ROM: Range of motion
SD: Standard deviation
TRA: Tibial roof angle
