# Supplementary material for: Long-term outcome (28–40 years) after correction of leg length discrepancy through permanent epiphysiodesis
Source: J Orthop Traumatol. 2025 Dec 16;26:76. doi: 10.1186/s10195-025-00895-2 (PMC12708432; doi:10.1186/s10195-025-00895-2)
Supplement: Supplementary file 1 — Additional file1 (DOCX 18 KB) [file 10195_2025_895_MOESM1_ESM.docx]

# Supplemental Tables

### *Supplemental Table I*

## **Oxford Knee Score Questionnaire Data of all Participants (n = 30)**

|  | Patients (n = 20) | Controls (n = 10) | *p*-value |
| --- | --- | --- | --- |
| OKS total score* |  |  | 0.003 |
| Median (IQR) | 46 (44-47) | 48 (47.8-48) |  |
| OKS pain† |  |  | 0.005 |
| Median (IQR) | 96 (89-200) | 100 (99-100) |  |
| OKS function† |  |  | 0.024 |
| Median (IQR) | 95 (90-100) | 100 (100-100) |  |

OKS: Oxford Knee Score; IQR: interquartile range.

*0 = Extreme problems, 48 = No problems

†0 = Extreme problems, 100 = No problems

## Supplemental Table II

## **EQ-5D-3L Questionnaire Data of all Participants (n = 30)**

| EQ-5D-3L Dimension* | Patients (n = 20) | Controls (n = 10) | *p*-value |
| --- | --- | --- | --- |
| Mobility *(no. of patients (%))* |  |  | 0.134 |
| Level 1 | 14 (73.7) | 10 (100) |  |
| Level 2 | 5 (26.3) | 0 (0) |  |
| Level 3 | 0 (0) | 0 (0) |  |
| Self-care *(no. of patients (%))* |  |  | 0.532 |
| Level 1 | 17 (89.5) | 10 (100) |  |
| Level 2 | 2 (10.5) | 0 (0) |  |
| Level 3 | 0 (0) | 0 (0) |  |
| Activities *(no. of patients (%))* |  |  | 0.268 |
| Level 1 | 14 (73.7) | 10 (100) |  |
| Level 2 | 4 (21.1) | 0 (0) |  |
| Level 3 | 1 (5.3) | 0 (0) |  |
| Pain *(no. of patients (%))* |  |  | 0.126 |
| Level 1 | 9 (47.4) | 8 (80) |  |
| Level 2 | 10 (52.6) | 2 (20) |  |
| Level 3 | 0 (0) | 0 (0) |  |
| Anxiety *(no. of patients (%))* |  |  | 1 |
| Level 1 | 16 (84.2) | 9 (90) |  |
| Level 2 | 3 (15.8) | 1 (10) |  |
| Level 3 | 0 (0) | 0 (0) |  |
| Health today† |  |  | 0.012 |
| Median (IQR) | 80 (70-86) | 90 (84-95) |  |

no.: number.

*Level 1 = No problems, Level 2 = Some problems, Level 3 = Extreme problems

†0 = Extreme problems, 100 = No problems

## Supplemental Table III

## **Knee Injury and Osteoarthritis Outcome Score Questionnaire Data of all Participants (n = 30)**

| KOOS Dimension* | Patients (n = 20) | Controls (n = 10) | *p*-value |
| --- | --- | --- | --- |
| Pain |  |  | 0.475 |
| Median (IQR) | 100 (94-100) | 100 (94-100) |  |
| Symptoms |  |  | 0.597 |
| Median (IQR) | 100 (96-100) | 100 (95-100) |  |
| Activities |  |  | 0.509 |
| Median (IQR) | 99 (96-100) | 100 (97-100) |  |
| Sport |  |  | 0.186 |
| Median (IQR) | 95 (90-100) | 100 (94-100) |  |
| Quality of life |  |  | 0.059 |
| Mean (SD) | 94 (16.2) | 100 (7.9) |  |
| Minimum | 44 | 75 |  |
| Maximum | 100 | 100 |  |

KOOS: Knee Injury and Osteoarthritis Outcome Score; IQR: interquartile range; SD: standard deviation.

*0 = Extreme problems, 100 = No problems
